# Dataset and protocols on the applicability of the BDM mechanism in product evaluation

**DOI:** 10.1016/j.dib.2019.104060

**Published:** 2019-05-25

**Authors:** Marcel Lichters, Verena Wackershauser, Shixing Han, Bodo Vogt

**Affiliations:** Otto-von-Guericke-University Magdeburg, P.O. Box 4120, 39106, Magdeburg, Germany

**Keywords:** Becker-DeGroot-Marschak (BDM) mechanism, Misconception bias, Product evaluation, Partial least squares (PLS), Willingness-to-pay (WTP)

## Abstract

Understanding how to best elicit consumers' willingness to pay (WTP) for goods in an incentive-aligned way is one of the cornerstones in marketing planning decisions and consumer welfare theory. This article provides a dataset from an experiment with n = 107 consumers that measured the WTP for a set of eight real consumer goods by means of the Becker-DeGroot-Marschak (BDM) mechanism, entailing a facultative resell option. This procedure allows for testing the empirical incentive-compatibility of the BDM mechanism. Despite early evidence on lottery choices or fictitious goods, the empirical incentive-compatibility of the BDM mechanism in case of real consumer goods remains an under-researched topic. For the first time, we provide a dataset on consumers' WTP statements in such a paradigm. More precisely, this article provides experimental protocols, manipulation-check questions, full raw datasets, summary statistics, and model properties related to the research article entitled “On the applicability of the BDM mechanism in product evaluation” [Lichters et al., 2019]. The raw dataset allows for an independent analysis of consumers’ bidding behavior for multiple consumer goods. Thus, future researchers might use our dataset in a meta-analytic fashion for their own research on WTP elicitation.

**Table:** Specifications table

Subject area	*Economics, Business Research*
More specific subject area	*Marketing, Retailing, Consumer Research, Consumer Behavior*
Type of data	*Text files, figures, tables, raw dataset*
How data was acquired	*The study drew on a computer-based experiment with 107 students from a major German university. All of them provided written consent for participation and were interested in the product categories under research. In the course of a pre-recruitment, each participant received a fee of €10 two weeks prior to study execution.*
Data format	Raw and analyzed data as *.csv, codebook as *txt file, figures
Experimental factors	*The study included four categories of consumer goods (electric toothbrushes, headphones, USB hard drives, and whisky); each covered a product with a low and a high category-specific market price. The eight products were presented in random order (within-subjects) to reduce other confounding effects.*
Experimental features	*The study's objective was to observe “irrational bidding behavior” (i.e., misconception bias) when applying the BDM mechanism*[1]*in a product evaluation context, evinced by the difference between a consumer's stated WTP p and the product's redemption price RP. Data analysis further covered a PLS-SEM model to gain a richer understanding on the drivers of misconception bias.* *Each participant (a) indicated a sufficient willingness to buy in the four product categories, (b) had prior buying experience within the four product groups, and (c) owned a certain aided brand awareness for all brands in the study. Participants worked on eight product-specific willingness to pay questions following the Becker-DeGroot-Marschak procedure. The study concluded with a facultative resell option (participants were free to resell the product at a given market price). Finally, participants provided further information about themselves (e.g., demographics).*
Data source location	*Magdeburg, Saxony-Anhalt, Germany*
Data accessibility	*Data is available with this article.*
Related research article	*Lichters, M., Wackershauser, V., Han, S., Vogt, B., 2019. On the applicability of the BDM mechanism in product evaluation. Journal of Retailing and Consumer Services, 51(6), 1–7. https://doi.org/10.1016/j.jretconser.2019.02.021.*

**Table:** 

**Value of the data** • This is the first open-access dataset that provides consumers' binding WTP statements on eight consumer goods, which were elicited by means of the Becker-DeGroot-Marschak (BDM) procedure with a facultative resell option. • The dataset enables a deeper understanding of consumer behavior in the elicitation of willingness to pay (WTP) according to a BDM mechanism that involves an a priori known buyback price. It allows for an analysis of the BDM mechanism's empirical incentive-compatibility for a broad set of consumer goods. • The dataset enables researchers to run their own analyses in an effort to learn more on consumers' bidding behavior in BDM elicitation tasks. • Future researchers might combine our dataset with their own data on other consumer goods in a meta-analytic fashion. • Considering the integral role of WTP elicitation in diverse fields such as marketing, retailing, economics, health economics, and consumer research, the dataset and descriptions will enable other researchers to set up comparable studies (the article may thus serve as a reference). • Finally, the dataset might inspire future researchers to work on methods that seek for an adjustment of consumers' biased WTP statements in BDM tasks with a resell option.

## 1 Data

**Fig. 1:**
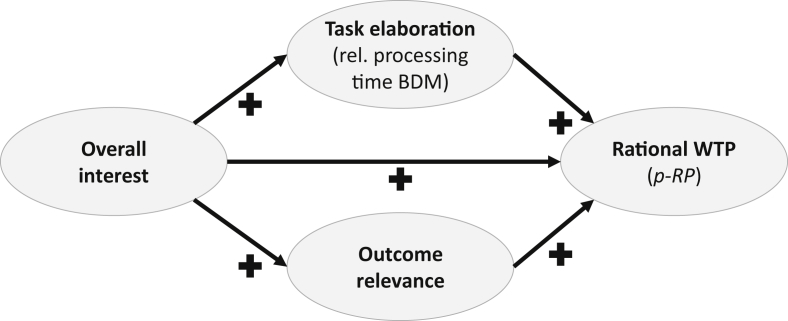
Proposed structural model that explains the extent to which individuals behave irrationally in a BDM willingness to pay elicitation with a resell option.

The provided dataset supplements the one described in Lichters et al. [1]. It stems from an experiment, which was conducted in Magdeburg (Germany) and incorporates information from 107 consumers. Specifically, the dataset covers socio-demographic characteristics, WTP statements for eight consumer goods following the BDM mechanism [2] with a facultative resell option and variables that operationalize the latent constructs *Rational WTP*, *Task elaboration*, *Outcome relevance*, and *Overall product interest*. Fig. 1 depicts the proposed structural model, which could be analyzed by independent researchers with the help of a Partial-Least-Squares Structural Equation Model (PLS-SEM) [3].

**Fig. 2:**
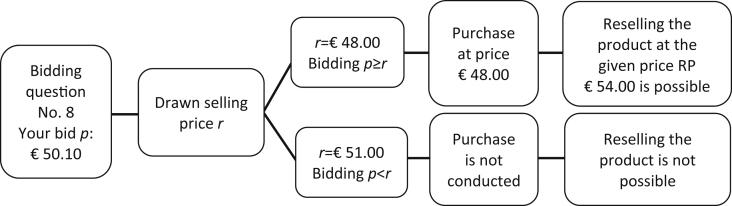
Illustration of the possible outcomes of the random payoff mechanism.

**Fig. 3:**
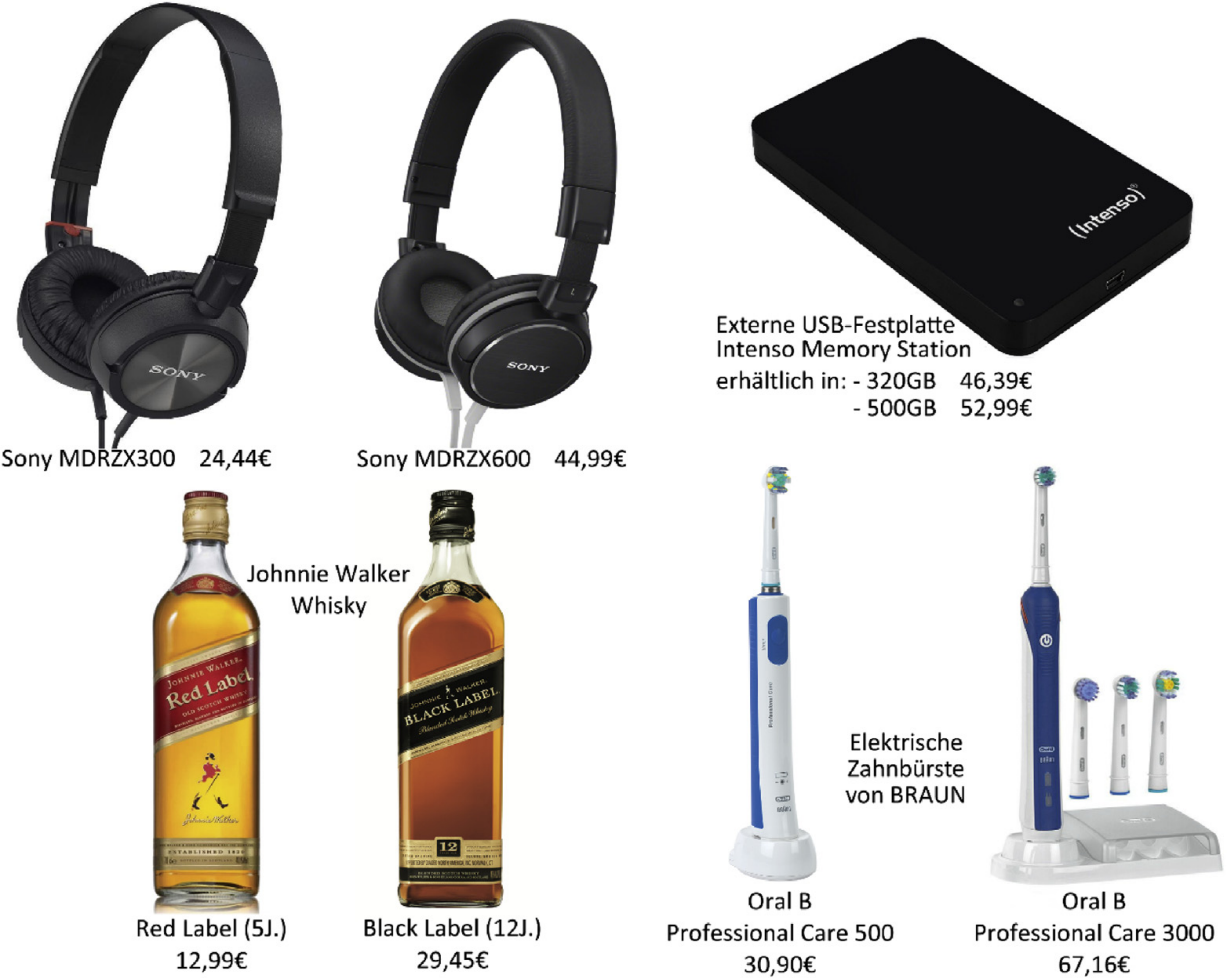
Products under research paired with their market prices as shown to the participants (German version).

Beyond that, Fig. 2 visualizes the random payoff mechanism implemented in the corresponding experiment, while Fig. 3 presents the eight consumer goods under research.

In addition, this article covers four tables, with the first (Table 1) reporting the operationalization (item wordings) and descriptive statistics for each of the focal variables in the dataset. Table 2 further presents the rotated matrix of component loadings resulting from a principal component analysis of the eight product-specific differences between the given resell price and the stated WTP. Besides, Table 3 includes statistics on the measurement model's reliability and validity. Finally, Table 4 reports the model's discriminant validity according to the Fornell-Larcker criterion as well as the heterotrait-monotrait ratio of correlations [4].

**Table 1:** Item wording (if question), mean, and standard deviation of the measured items.

Construct	Item	Label/Operationalization	Mean	SD
Overall interest^a^	1	Interest in buying Oral-B Professional Care 500	2.13	1.61
	2	Interest in buying Oral-B Professional Care 3000	1.70	1.38
	3	Interest in buying Johnnie Walker Red Label	2.86	2.00
	4	Interest in buying Johnnie Walker Black Label	2.56	1.92
	5	Interest in buying Intenso Memory Station 320 GB	2.41	1.81
	6	Interest in buying Intenso Memory Station 500 GB	2.76	2.03
	7	Interest in buying Sony MDRZX300	2.92	2.04
	8	Interest in buying Sony MDRZX600	2.53	1.84
Outcome relevance^b^	1	Excitement: How excited are you about seeing the outcomes of the random payoff lottery?	4.52	1.88
	2	Deal Proneness: How intensely do you hope to receive a product at a special bargain price as a result of the experiment?	5.23	1.92
Task elaboration	1	Rel. processing time in BDM task regarding Oral-B Professional Care 500	0.16	0.08
	2	Rel. processing time in BDM task regarding Oral-B Professional Care 3000	0.09	0.08
	3	Rel. processing time in BDM task regarding Johnnie Walker Red Label	0.08	0.05
	4	Rel. processing time in BDM task regarding Johnnie Walker Black Label	0.07	0.06
	5	Rel. processing time in BDM task regarding Intenso Memory Station 320 GB	0.10	0.07
	6	Rel. processing time in BDM task regarding Intenso Memory Station 500 GB	0.08	0.09
	7	Rel. processing time in BDM task regarding Sony MDRZX300	0.09	0.07
	8	Rel. processing time in BDM task regarding Sony MDRZX600	0.09	0.07
Rational WTP (€)	1	Deviation from optimal WTP (p-RP): Oral-B Professional Care 500	−16.49	10.21
	2	Deviation from optimal WTP (p-RP): Oral-B Professional Care 3000	−41.97	22.16
	3	Deviation from optimal WTP (p-RP): Johnnie Walker Red Label	−5.89	4.37
	4	Deviation from optimal WTP (p-RP): Johnnie Walker Black Label	−15.32	9.74
	5	Deviation from optimal WTP (p-RP): Intenso Memory Station 320 GB	−25.32	15.23
	6	Deviation from optimal WTP (p-RP): Intenso Memory Station 500 GB	−27.15	17.73
	7	Deviation from optimal WTP (p-RP): Sony MDRZX300	−11.71	8.01
	8	Deviation from optimal WTP (p-RP): Sony MDRZX600	−23.93	14.95

a 7-point scale: from +1 (not at all interested) to +7 (very interested).

b 7-point scale: from +1 (not at all) to +9 (very).

**Table 2:** Rotated component matrix of a principal component analysis based on the individual *p-RP* differences.

Product	Number of component			
	1	2	3	4
Braun Oral-B Professional Care 500	.882			
Braun Oral-B Professional Care 3000	.694			
Johnnie Walker Red Label		.947		
Johnnie Walker Black Label		.684		
Intenso Memory Station 320 GB			.818	
Intenso Memory Station 500 GB			.843	
Sony MDRZX300				.880
Sony MDRZX600				.711
Variance explained (Σ = 91.41%)	22.48%	20.13%	26.16%	22.65%

Remark: Loadings < 0.5 are not shown.

**Table 3:** Evaluation of the measurement model including assessment of internal consistency, convergent validity, loadings, and their significance (one-tailed).

Construct/Indicator	Item	Loading	p-Value
**Overall interest** (formative)			
Braun Oral-B Professional Care 500	1	0.373	0.003
Braun Oral-B Professional Care 3000	2	0.347	0.001
Johnnie Walker Red Label	3	0.497	0.000
Johnnie Walker Black Label	4	0.524	0.000
Intenso Memory Station 320 GB	5	0.494	0.000
Intenso Memory Station 500 GB	6	0.629	0.000
Sony MDRZX300	7	0.746	0.000
Sony MDRZX600	8	0.593	0.000
**Outcome relevance** (reflective, AVE = 0.798, α = 0.748, ρ = 0.888)			
Interest in the results of the lottery procedure	1	0.900	0.000
Interest to make a special bargain as the result of the study	2	0.887	0.000
**Task elaboration** (reflective, AVE = 0.332, α = 0.719, ρ = 0.788)			
Processing time: Braun Oral-B Professional Care 500	1	0.397	0.016
Processing time: Braun Oral-B Professional Care 3000	2	0.569	0.001
Processing time: Johnnie Walker Red Label	3	0.662	0.000
Processing time: Johnnie Walker Black Label	4	0.665	0.000
Processing time: Intenso Memory Station 320 GB	5	0.570	0.002
Processing time: Intenso Memory Station 500 GB	6	0.268	0.055
Processing time: Sony MDRZX300	7	0.560	0.000
Processing time: Sony MDRZX600	8	0.764	0.000
**Rational WTP (Deviation of*p*from optimal WTP RP)** (reflective, AVE = 0.670, α = 0.927, ρ = 0.941)			
Braun Oral-B Professional Care 500	1	0.789	0.000
Braun Oral-B Professional Care 3000	2	0.890	0.000
Johnnie Walker Red Label	3	0.569	0.000
Johnnie Walker Black Label	4	0.824	0.000
Intenso Memory Station 320 GB	5	0.882	0.000
Intenso Memory Station 500 GB	6	0.874	0.000
Sony MDRZX300	7	0.797	0.000
Sony MDRZX600	8	0.876	0.000

**Table 4:** The reflectively measured constructs’ discriminant validity (5000 bootstrap samples).

Fornell-Larcker criterion/heterotrait-monotrait ratio of correlations (95% C.I.’s)			
	Outcome relevance	Task elaboration	Rational WTP
Outcome relevance	0.893	[0.120 | 0.286]	[0.486 | 0.730]
Task elaboration	0.082	0.576	[0.284 | 0.495]
Rational WTP	0.512	0.355	0.819

## 2 Experimental design, materials and methods

### 2.1 Experimental design

The dataset is based on a computerized experiment with 107 German consumers. In the course of a pre-recruitment, each participant received a fee of €10 two weeks prior to the experiment's sessions to suppress house money effects [5].

The investigation included four categories of consumer goods (electric toothbrushes, headphones, USB hard drives, and whisky); each of the categories thereby covered a product with a low and a high category-specific market price. The eight products were presented in random order (within-subjects). All participants further had the opportunity to evaluate the relevant products from a product shelf without prices [6] prior to their WTP statements.

At the beginning of the computerized interview, participants indicated their (a) individual willingness to buy in the four product categories [7], (b) prior buying experience within the four product groups, and (c) aided brand awareness for all brands under research [8].

Subsequently, the BDM mechanism and the resell option were introduced personally and, once again, on the computer screens. The participants were further informed about the implemented random payoff mechanism (RPM), which would draw one out of all BDM tasks to become payoff-relevant [9].

In order to check whether all participants understood the BDM, the RPM and the resell functioning correctly, we required them to respond to two multiple-choice questions [10]. The multiple-choice questions used the following wording:

*The following part of the survey explains how the purchase will* *(or will not)* *be generated* *based on* *the mechanism. In the subsequent purchase decisions, we will ask you to submit a bid price for the products shown. This bid price represents the highest price you are willing to pay for the product at this specific moment. Among all eight presented purchase decisions, we will randomly render one to become relevant at the end of the study. The question, at present, is whether a purchase decision will be conducted. The mechanism proceeds as follows:*

1. *A random selling price r will be drawn for the product.*
2. *The drawn selling price* *r* *will then be compared with your bid price p.*
3. *If the selling price is less than or equal to your bid p, the purchase will be conducted and you will pay the selling price r. If, however, the selling price is greater than your bid, you will not be able to buy the product.*

*An example: We render the bidding in question No. 8 as the relevant purchase decision. We then consider your bid p for the product in question No. 8. Your bid p for the product is* *€* *50.10. Subsequently, you pull the selling price r from an urn.*

*The randomly drawn selling price r is* *€* *48.00. Therefore, you buy the product at a price of* *€* *48.00, because* *€* *48.00 is less than your bid p of* *€* *50.10. However, if you had drawn a selling price of* *€* *51.00, no purchase transaction would have taken place.*

*Please note**: In any case, it is to your advantage to actually bid the highest price you are willing to pay at the moment. Otherwise, there is a risk that you will not be able to buy a product, although you would have liked to buy the product at the drawn price r.*

*Please note further**: If a purchase decision from this survey section becomes relevant to you, we will also offer you a buyback option. This means that you will be able to resell the product immediately. The repurchase price RP at which you will be able to resell the product is represented by the actual market price in the respective purchase situation. Please note that you can only decide whether or not to resell the product at RP after a successful purchase* (see Fig. 2) *.*

*For example, your bid p for the product in the relevant purchase decision No. 8 is* *€* *50.10 and the drawn selling price r turns out to be* *€* *48.00. Consequently, you have to pay* *€* *48.00 for the product in exchange. Afterwards, it is your choice to decide whether or not to resell the product at RP, which is* *€* *54.00 in this example. If your draw had been a selling price of* *€* *51.00, no purchase transaction would have been taken place. Thus, in this case, you would not have been offered the option to resell the product at RP.*

*Multiple-choice question1*. *Before we continue with the study, imagine the following situation:*

*Josef Jedermann participates in a similar study. In one of his purchase decisions, he is offered a high-quality espresso machine. The proposed repurchase price RP is* *€* *599.00. His bidding price p for the espresso machine is* *€* *579.00. After all bidding rounds, his decision in this situation becomes relevant. He now draws the selling price r of* *€* *569.00.*

*What are the consequences for Josef?*

a) *He cannot buy the espresso machine.*
b) *He can buy the espresso machine and pays* *€* *579.00. Furthermore, he can consider reselling the machine at* *€* *599.00.*
c) *He can buy the espresso machine and pays* *€* *569.00. Furthermore, he can consider reselling the machine at* *€* *599.00.*
d) *He can buy the espresso machine and pays* *€* *569.00. Furthermore, he cannot consider reselling the machine.*

*Multiple-choice question2*. *Now imagine, Max Mustermann participates in the same survey.*

*He also wants to buy a high-quality espresso machine. The proposed repurchase price is* *€* *165.00. His price offer is* *€* *179.00. He draws a selling price r of* *€* *180.00. What are the consequences for Max?*

a) *He cannot buy the espresso machine.*
b) *He can buy the espresso machine at* *€* *179.00. Furthermore, he can consider reselling the machine at* *€* *165.00.*
c) *He can buy the espresso machine at* *€* *180.00. Furthermore, he can consider reselling the machine at* *€* *165.00.*

After having responded to both multiple-choice questions, all participants worked on eight product-specific WTP questions following the BDM mechanism. Each provided a facultative resell option at the actual market price (*RP*) of the focal item. Thus, each participant was expected to state at least *RP* as WTP. In a final step, all participants further provided information about themselves (e.g., demographics).

### 2.2 Materials

Fig. 3 presents all eight implemented products and their corresponding market prices in €.

### 2.3 Operationalization of the focal variables

The accompanying dataset contains the following variables that were used for reflectively operationalizing the endogenous constructs in Fig. 1:

• *Rational WTP*: eight indicators [11], one for each difference calculated by the reported WTP (*p*) minus the optimal WTP (the product's specific market price, which was offered as the resell price [*RP*]).
• *Task elaboration*: task-specific relative processing times; specifically, each relative processing time value represents the time a participant took to complete a specific BDM task relative to the total time he/she needed to complete all the other, unrelated questions (i.e., demographics, *Overall product interest*, *Outcome relevance*).
• *Outcome relevance*: two questions; the first seeks for an assessment to which extent a participant is interested in making a profitable deal as the result of the experiment (“How intensely do you hope to receive a product at a special bargain price as a result of the experiment? ”; following items from [12] on pleasure in bargains). The second question relates to the excitement about the outcome of the random payoff mechanism [13] (“How excited are you about seeing the outcomes of the random payoff lottery?”).

Moreover, *Overall product interest* was a formatively operationalized construct with product-specific (not necessarily correlated) interest ratings as indicators.

Table 1 presents the item wordings along with descriptive statistics for all variables in the model.

### 2.4 Overview over the model properties

Table 2 presents the component loadings, which stem from a principal component analysis of individual differences between the stated *p* and the *RP* (with four components forced to be extracted, and a Varimax rotation).

Table 3 displays all statistics, which are commonly used to assess the quality of the measurement model.

Table 4 further reports the model's discriminant validity according to the Fornell-Larcker criterion as well as the heterotrait-monotrait ratio of correlations [4], [14].

## References

[1] Lichters M., Wackershauser V., Han S., Vogt B. (2019). On the applicability of the BDM mechanism in product evaluation. J. Retail. Consum. Serv..

[2] Becker G.M., Degroot M.H., Marschak J. (1964). Measuring utility by a single-response sequential method. Behav. Sci..

[3] Lohmöller J.B. (1989). Latent Variable Path Modeling with Partial Least Squares.

[4] Henseler J., Ringle C.M., Sarstedt M. (2015). A new criterion for assessing discriminant validity in variance-based structural equation modeling. J. Acad. Mark. Sci..

[5] Cox J.C., Kroll E.B., Lichters M., Sadiraj V., Vogt B. (2019). The St. Petersburg paradox despite risk-seeking preferences: an experimental study. Bus. Res..

[6] Lichters M., Sarstedt M., Vogt B. (2015). On the practical relevance of the attraction effect: a cautionary note and guidelines for context effect experiments. AMS Rev..

[7] Voelckner F. (2006). An empirical comparison of methods for measuring consumers' willingness to pay. Mark. Lett..

[8] Lichters M., Müller H., Sarstedt M., Vogt B. (2016). How durable are compromise effects?. J. Bus. Res..

[9] Ortega D.L., Wolf C.A. (2018). Demand for farm animal welfare and producer implications: results from a field experiment in Michigan. Food Policy.

[10] Wertenbroch K., Skiera B. (2002). Measuring consumers' willingness to pay at the point of purchase. J. Mark. Res..

[11] Irwin J.R., McClelland G.H., McKee M., Schulze W.D., Norden N.E. (1998). Payoff dominance vs. Cognitive transparancy in decision making. Econ. Inq..

[12] Mooradian T.A., Olver J.M. (1996). Shopping motives and the five factor model: an integration and preliminary study. Psychol. Rep..

[13] Dong S., Ding M., Huber J. (2010). A simple mechanism to incentive-align conjoint experiments. Int. J. Res. Mark..

[14] Girard A., Lichters M., Sarstedt M., Biswas D. (2019). Short- and long-term effects of nonconsciously processed ambient scents in a servicescape: findings from two field experiments. J. Serv. Res..

