# Supplementary material for: Dataset and protocols on the applicability of the BDM mechanism in product evaluation
Source: Data Brief. 2019 May 25;25:104060. doi: 10.1016/j.dib.2019.104060 (PMC6562191; doi:10.1016/j.dib.2019.104060)
Supplement: Multimedia component 1 [file mmc1.pdf]

Author Declaration

Data in Brief Manuscript: DIB-D-19-00522

Title: Data and analyses on the applicability of the BDM mechanism in product evaluation

Conflict of Interest and Authorship Conformation Form Please check the following as appropriate:

- [x] All authors have participated in (a) conception and design, or analysis and interpretation of the data; (b) drafting the article or revising it critically for important intellectual content; and (c) approval of the final version.

- [x] This manuscript has not been submitted to, nor is under review at, another journal or other publishing venue.

- [x] The authors have no affiliation with any organization with a direct or indirect financial interest in the subject matter discussed in the manuscript.

We wish to confirm that there are no known conflicts of interest associated with the publication and there has been no significant financial support for this work that could have influenced its outcome.

We confirm that there are no other persons who satisfied the criteria for authorship but are not listed. We further confirm that the order of authors listed in the manuscript has been approved by all of us.

We confirm that we have given due consideration to the protection of intellectual property associated with this work and that there are no impediments to publication, including the timing of publication, with respect to intellectual property. In so doing we confirm that we have followed the regulations of our institutions concerning intellectual property.

We understand that the Corresponding Author is the sole contact for the Editorial process (including Editorial Manager and direct communications with the office). He is responsible for communicating with the other authors about progress, submissions of revisions and final approval of proofs. We confirm that we have provided a current, correct email address which is accessible by the Corresponding Author and which has been configured to accept email from Data in Brief. The email address is Marcel.Lichters@ovgu.de

Signed by all authors as follows:

Marcel Lichters (corresponding author): ____________, date: 21/03/2019

Verena Wackershauser: ____________, date: 21/03/2019

Shixing Han: ____________, date: 21/03/19

Bodo Vogt: ____________, date: 21/03/19
